# San-Huang-Yi-Shen Capsule Ameliorates Diabetic Kidney Disease through Inducing PINK1/Parkin-Mediated Mitophagy and Inhibiting the Activation of NLRP3 Signaling Pathway

**DOI:** 10.1155/2022/2640209

**Published:** 2022-11-15

**Authors:** Hanzhou Li, Yuansong Wang, Xiuhai Su, Qinghai Wang, Shufang Zhang, Wenjuan Sun, Tianyu Zhang, Mengxue Dong, Zhaiyi Zhang, Shuquan Lv

**Affiliations:** ^1^Chengde Medical University, Chengde, China; ^2^Cangzhou Hospital of Integrated Traditional Chinese Medicine and Western Medicine of Hebei Province, Cangzhou, China; ^3^Tianjin University of Traditional Chinese Medicine, Tianjin, China

## Abstract

San-Huang-Yi-Shen capsule (SHYS) has been used in the treatment of diabetic kidney disease (DKD) in clinics. However, the mechanism of SHYS on DKD remains unclear. In this study, we used a high-fat diet combined with streptozocin (STZ) injection to establish a rat model of DKD, and different doses of SHYS were given by oral gavage to determine the therapeutic effects of SHYS on DKD. Then, we studied the effects of SHYS on PINK1/Parkin-mediated mitophagy and the activation of NLRP3 inflammasome to study the possible mechanisms of SHYS on DKD. Our result showed that SHYS could alleviate DKD through reducing the body weight loss, decreasing the levels of fasting blood glucose (FBG), and improving the renal function, insulin resistance (IR), and inhibiting inflammatory response and oxidative stress in the kidney. Moreover, transmission electron microscopy showed SHYS treatment improved the morphology of mitochondria in the kidney. In addition, western blot and immunoflourescence staining showed that SHYS treatment induced the PINK1/Parkin-mediated mitophagy and inhibited the activation of NLRP3 signaling pathway. In conclusion, our study demonstrated the therapeutic effects of SHYS on DKD. Additionally, our results indicated that SHYS promoted PINK1/Parkin-mediated mitophagy and inhibited NLRP3 inflammasome activation to improve mitochondrial injury and inflammatory responses.

## 1. Introduction

Diabetic kidney disease (DKD), one of the leading diabetic complications, is a chronic structural and functional kidney disorder [1]. Early DKD usually manifests as a progressive decrease in glomerular filtration rate and elevation in serum creatinine, microalbuminuria, and an eventual progression to macroalbuminuria. Moreover, DKD can progress to end-stage renal failure if not promptly treated [2]. Modern medicine usually treats DKD through controlling glucose and blood pressure, reducing inflammation and oxidative stress, and improving microcirculation [3]. However, these treatments cannot inhibit the progression of DKD, and some hypotensive drugs, such as angiotensin-converting enzyme inhibitors, can cause dry cough, abnormal blood potassium, and other adverse reactions [4]. Therefore, it is critical to identify feasible early intervention measures to block or delay the progression of DKD.

Many studies have shown that traditional Chinese medicine (TCM) and its active ingredients have significant advantages in improving renal function and alleviating inflammation in DKD. A clinical meta-analysis found that Yu-Quan pills combined with Western medicine can significantly decrease proteinuria, serum creatinine (Cr), and blood urea nitrogen (BUN) levels in patients with DKD [5]. Salidroside can regulate the Sirt1/PGC-1*α* axis in renal tissues, thereby regulating mitochondrial biosynthesis and alleviating renal impairment in a mouse DKD model [6]. In rats with DKD, curcumin can upregulate autophagy to inhibit the mesenchymal transdifferentiation in podocytes [7]. Huang-Kui capsules can inhibit the activation of NLRP3 and TLR4/NF-*κ*B pathways, improving the epithelial–mesenchymal transition in the renal tubule in DKD rats [8]. Therefore, elucidating the mechanisms of TCM on DKD could provide the scientific evidence of TCM on DKD and contribute to the clinical use of TCM to treat DKD.

San-Huang-Yi-Shen capsule (SHYS) consists of *Astragalus membranaceus*, *Panax quiquefolium* L., Rhizoma Dioscoreae, *Cornus officinalis*, Semen Cuscutae, *Rhizoma polygonati*, *Rehmannia glutinosa* Libosch, Semen Euryales, *fructus rosae laevigatae*, *Leonurus sibiricus* L., *Salvia miltiorrhiza*, *Ligusticum wallichii*, *Rhizoma atractylodis*, Radix Paeoniae Rubra, and Cowherb Seed is widely used in the treatment of DKD and other chronic nephropathies. Our previous clinical study found that SHYS could significantly improve renal function and delay the progression in patients with DKD [9]. However, the mechanisms by which SHYS improves DKD are still unknown.

In this study, we used a high-fat diet (HFD) combined with streptozocin (STZ) injection to establish a rat model of DKD, and different doses of SHYS were given by oral gavage to determine the therapeutic effects of SHYS on DKD. Then, we studied the effects of SHYS on PINK1/Parkin-mediated mitophagy and the activation of NLRP3 inflammasome to study the possible mechanisms of SHYS on DKD.

## 2. Methods

### 2.1. Materials and Methods

HFD (10% lard, 20% sucrose, 2.5% cholesterol, and 67.5% normal feed) was purchased from Beijing Sibeifu Bioscience Co., Ltd. (Beijing, China). Streptozotocin (STZ) was purchased from Solarbio Biotechnology Co., Ltd. (Beijing, China). All biochemical test kits (creatinine (Cr), blood urea nitrogen (BUN), urea protein, superoxide dismutase (SOD), methane dicarboxylic aldehyde (MDA), and glutathione peroxidase (GSH-Px)) were obtained from Nanjing Jiancheng Biological Engineering Institute (Nanjing, China). Enzyme-linked immunosorbent assay (ELISA) kits of rat insulin, tumor necrosis factor alpha (TNF-*α*), and interleukin (IL)-1*β*, IL-6 were purchased from Multi Sciences Biotechnology Co., Ltd. (Hangzhou, China). VDAC1 monoclonal antibody (66345-1-Ig), TOM20 polyclonal antibody (11802-1-AP), COXIV polyclonal antibody (11242-1-AP), LC3 polyclonal antibody (14600-1-AP), p62 polyclonal antibody (18420-1-AP), PINK1 polyclonal antibody (23274-1-AP), Parkin monoclonal antibody (66674-1-Ig), *β*-actin polyclonal antibody (20536-1-AP, HRP-conjugated affinipure goat anti-rabbit IgG(H + L) (SA00001-2), and HRP-conjugated affinipure goat anti-mouse IgG(H + L) (SA00001-1) were purchased from Proteintech, Inc. (Wuhan, China). Rabbit anti-NLRP3 antibody (bs-10021R) and rabbit anti-ASC antibody (bs-6741R) were obtained from Bioss Bioscience Co., Ltd. (Beijing, China). Anti-IL-1 beta antibody (ab283818), anti-caspase-1 antibody (ab207802) and anti-IL-18 antibody (ab191860) were obtained from Abcam, Inc. (Shanghai, China).

### 2.2. Preparation of SHYS

SHYS was prepared from the pharmacy department of Cangzhou Hospital of Integrated Traditional Chinese and Western Medicine. Briefly, 15 g of *Astragalus membranaceus*, 12 g of *Panax quiquefolium L.*, 12 g of *Rhizoma Dioscoreae*, 12 g of *Cornus officinalis*, 12 g of Semen Cuscutae, 12 g of *Rhizoma polygonati*, 12 g of *Rehmannia glutinosa* Libosch, 12 g of Semen Euryales,12 g of Fructus Rosae Laevigatae,12 g of *Leonurus sibiricus* L., 12 g of *Salvia miltiorrhiza*, 12 g of *Ligusticum wallichii*, 10 g of *Rhizoma atractylodis*, 10 g of Radix Paeoniae Rubra, and 6 g of Cowherb Seed were weighed and capsuled according to the medical institution preparation standard in Hebei (approval number: Z20050795).

### 2.3. Model Construction and Dosing

Forty healthy, 6–8-week-old, specific pathogen free- (SPF-) grade male Sprague-Dawley (SD) rats weighing 200 ± 20 g were provided by Beijing Huafukang Biotechnology Co., Ltd. (production approval number: SCXK (Beijing) 2019-0008). Their housing environment was maintained at 25°C ± 2°C, the relative humidity was 50% ± 15%, the light-dark cycle was 12 h/12 h, and the animals were given ad libitum access to food and water. This experiment was approved by the ethics committee of Cangzhou Hospital of Integrated Traditional Chinese Medicine and Western Medicine (Approval no.: CZX2021-KY-023).

After the rats were acclimatized for 1 week, 10 rats were randomly selected for the control group and housed in a routine, clean-grade facility. The remaining 30 rats were given HFD. After they were given the special feed for 7 weeks, STZ 30 mg/kg [1% STZ solution prepared using 0.1 mmol/L citric acid-sodium citrate buffer (pH = 4.4)] was given by intraperitoneal injection. Animals were fasted for 12 h before the injection but were allowed to drink water. After 72 h, blood was collected from the tail vein to check if blood glucose was ≥16.7 mmol/L, which was used to confirm that the diabetic rat model was successfully constructed. Diabetic rats were housed for another 1 week. After 1 week, the rats were housed in metabolic cages and we performed 24 h of urine protein quantitation. Blood glucose level ≥ 16.7 mmol/L and urine protein ≥20 mg/24 h were used as criteria for the successful induction of the DKD animal model [10]. One week after STZ injection, the DKD model was successfully induced (Figure S1).

DKD rats were divided into the model group, the SHYS low-dose group, and the SHYS high-dose group, with 10 rats per group. The control group and the model group were given an equal volume of distilled water via intragastric administration. Meanwhile, 0.81 g/kg and 1.62 g/kg of SHYS were given via intragastric administration for 8 continuous weeks to the SHYS low-dose group and the SHYS high-dose group, respectively. The dosages of SHYS were calculated based on human–rat body surface area conversion. During the study, rats were given ad libitum access to water and a standard diet. Fasting blood glucose (FBG) and body weight was measured every two weeks during SHYS treatment.

### 2.4. Biochemical Markers

After 8 weeks of SHYS intervention, metabolic cages were used to collect urine output from the groups, which was then centrifuged at 3000 rpm for 10 minutes. The urine protein content was measured according to the manufacturer's instructions.

Besides, sodium pentobarbital (50 mg/kg) was administered by intraperitoneal injection to anesthetize the rat, and inner canthus blood was collected. The blood was centrifuged at 3,000 rpm for 15 minutes to collect serum. A fully automatic biochemical analyzer measured serum Cr and BUN levels in the various groups.

In addition, 100 mg of renal tissues were weighed and immersed into 900 *μ*L of normal saline and homogenized on ice. Then, the homogenized renal tissue mixture was centrifuged at 3,000 rpm for 15 min and the supernatant was collected to obtain the tissue homogenate. The activities of SOD and GSH-Px and the level of MDA were investigated following the established protocol of test kits. The total protein contents in tissue homogenates were quantified using BCA protein concentration test kits.

### 2.5. ELISA

The fasting insulin (FINS) level in serum and the levels of proinflammatory cytokines (IL-1*β*, IL-6 and TNF-*α*) in tissue homogenates were measured using ELISA based on the established protocol of the kits. The IR was evaluated based on homeostatic model assessment of IR (HOMA-IR) using the following formula: HOMA − IR = FBG × FINS/22.5. Besides, the total protein content in tissue homogenates were quantified using BCA protein concentration test kits.

### 2.6. Renal Tissue Histopathological Staining

Renal tissues from the groups were fixed with a 10% formalin solution for 24 h, washed with water for 20 minutes, followed by dehydration using an alcohol gradient, xylene cleared, and then paraffin-embedded. Five *μ*m sections were cut and used for the hematoxylin and eosin (HE), Masson, and Sirius red staining. Pathological changes in rat renal tissues were observed under a microscope. The pathological changes in HE staining were evaluated based on the HE staining score as described previously [11] (Table S1). The collagen deposition in the Masson and Sirius red staining was quantified based on the average optical density (AOD) using Image-Pro Plus 6.0.

In addition, fresh renal tissues were cut into 2 mm × 2 mm × 2 mm and immersed in 2.5% glutaraldehyde and dehydrated. The morphology of mitochondria was observed using a transmission electron microscope. The percentage of fragmented mitochondria was calculated .

### 2.7. Immunofluorescence

Renal tissues were fixed with formalin solution, embedded in paraffin, and cut into 3 *μ*m sections. This was followed by clearing and hydrating before antigen retrieval. Sections were permeabilized using 0.5% Triton X-100 for 5 min, then blocked with 5% BSA, and finally incubated at 37°C for 1 hour. The primary antibodies LC3 (1 : 100), LAMP2 (1 : 500), Parkin (1 : 500), and VDAC1 (1 : 500) were incubated at 4°C overnight. The corresponding secondary antibodies were added after washing with PBS three times, followed by DAPI counterstaining for 10 minutes. The sections were then washed with PBS three times and sealed with an antiquenching agent. Images were taken using an immunofluorescence microscope. Image Pro Plus 6.0 was used to quantitate the fluorescence intensity, total area, and mean optical density. The positive area was calculated based on the ratio between mean optical density and total area.

### 2.8. Western Blot

Rat renal tissues were lysed on ice for 30 minutes with RIPA lysis buffer. Samples were centrifuged at 12,000 rpm at 4°C for 10 minutes, and the supernatant was transferred to fresh EP tubes. BCA protein concentration test kit was used to measure the protein concentration in the supernatant. A 20 *μ*g sample was added to a 10% SDS-polyacrylamide gel for electrophoresis. The target proteins resolved in the gel were transferred to methanol-activated PVDF membranes. Membranes were blocked in 5% skim milk at room temperature for 2 hours before the antibodies, anti-VDAC1 (1 : 10,000), TOM20 (1 : 20,000), COXIV (1 : 20,000), LC3 (1 : 1,000), p62 (1 : 5,000), PINK1 (1 : 500), Parkin (1 : 5,000), NLRP3 (1 : 1,000), IL-1*β* (1 : 1,000), caspase-1 (1 : 1,000), ASC (1 : 500), IL-18 (1 : 500), and *β*-actin (1 : 5,000), were added and incubated at 4°C overnight. On Day 2, 1 × TBST buffer was used to wash the membrane three times for 10 minutes each. The corresponding secondary antibodies were added, and the membrane was incubated at room temperature for 2 h. 1 × TBST buffer was used to wash the membrane three times, 5 minutes each time. The enhanced chemiluminescence method was used to visualize the bands, and *β*-actin was the internal control. A gel imaging system was used for imaging. Image J software was used to analyze the gray values of each band to calculate the relative protein expression.

### 2.9. Statistical Analysis

The SPSS Statistics 20.0 statistical software was used to analyze the experimental results. Quantitative data are expressed as mean ± standard deviation, and a *t*-test was used for comparison. One-way ANOVA followed by Tukey's post-hoc test was used for multigroup comparison of means. A difference of *P* < 0.05 was considered to be statistically significant.

## 3. Results

### 3.1. Therapeutic Effects of SHYS on Rats with DKD

**Figure 1:**
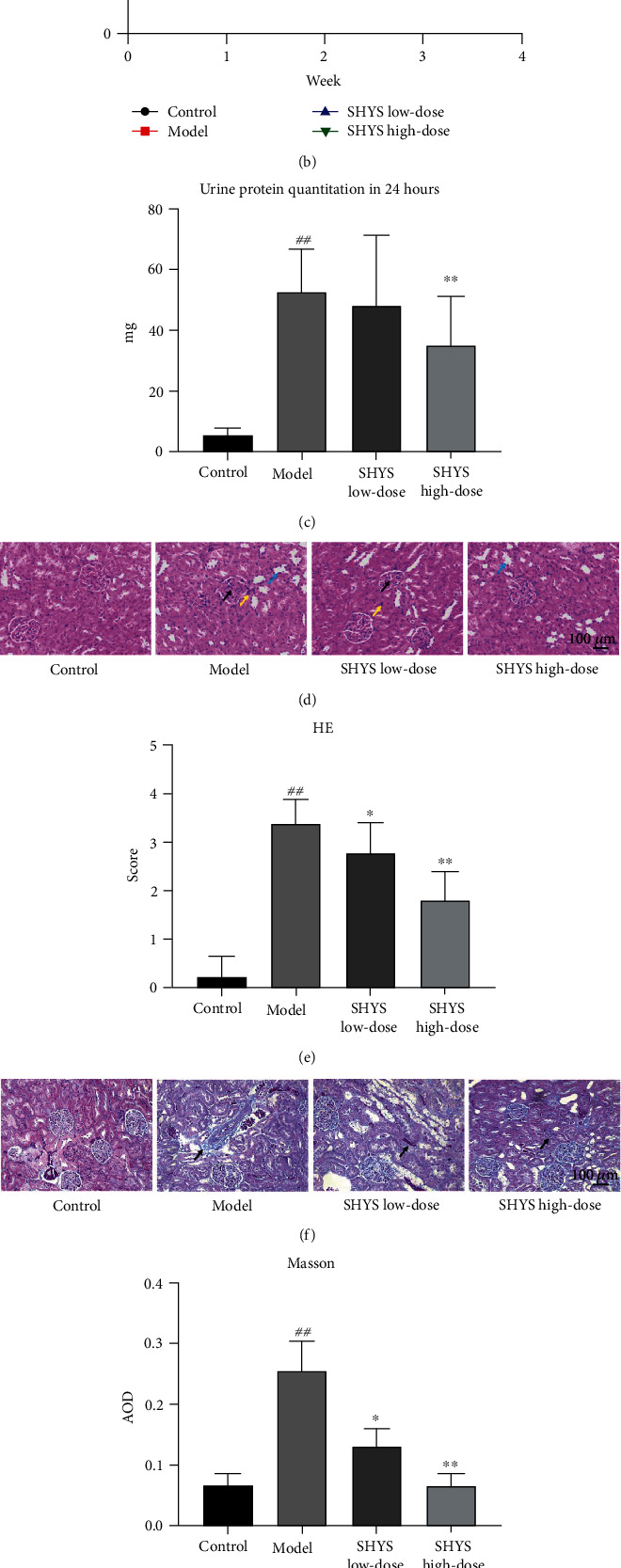
Therapeutic effects of SHYS on DKD model rats. DKD rats were orally treated with different dosages of SHYS, the therapeutic effects of SHYS on DKD were evaluated through measuring the body weight change, FBG, level of 24 h urine protein, and pathological changes in the kidney. (a) SHYS treatment reduced the body weight loss in DKD model rats. (b) SHYS treatment decreased the FBG level in DKD model rats. (c) SHYS treatment reduced the level of 24 h urine protein in DKD model rats. (d, e) HE staining showed that the pathological changes in the kidney in DKD model rats were improved after SHYS treatment (black arrows indicated the glomerular hyperplasia; blue arrows indicated the renal tubule atrophy; yellow arrows indicated the inflammatory cell infiltration) (200×) (d). SHYS treatment also lowered the HE score of kidney injury in rats with DKD (e). (f)–(i) The Masson (f, g) and Sirius red (h, i) staining showed that SHYS treatment decreased the accumulation of collagen contents in kidney (black arrows indicated the positive expression area) (200×). Control, model, SHYS low-dose, and SHYS high-dose groups (*n* = 10 per group) ^##^: *P* < 0.01 compared with the control group; ^∗^: *P* < 0.05 compared with the model group; ^∗∗^: *P* < 0.01 compared with the model group.

The body weight in the model group was lower and the FBG was higher in the model group compared with the rats in the control group (*P* < 0.01, respectively). Low-dose and high-dose of SHYS treatment reduced the body-weight loss (*P* < 0.05, *P* < 0.01, respectively) and decreased the FBG level (*P* < 0.01, respectively) in DKD model rats (Figures 1(a) and 1(b)). In addition, the serum levels of Cr and BUN and the levels of 24 h urine protein were higher in the model group compared with the control group (*P* < 0.01, respectively) whereas the Cr level was decreased in SHYS low-dose and SHYS high-dose groups (*P* < 0.05, *P* < 0.01, respectively) and the level of BUN was lower in the SHYS high-dose group (*P* < 0.05) compared with the model group (Table 1). High-dose of SHYS treatment reduced the levels of 24 h urine protein in DKD model rats (*P* < 0.01, Figure 1(c)). The HOMA-IR was higher in the model group compared with the control group (*P* < 0.01), low-dose and high-dose of SHYS treatment lowered the HOMA-IR in rats with DKD (*P* < 0.01, respectively, Table 2).

In addition, HE staining showed significant degeneration and atrophy in renal tubulars, mesangial hyperplasia in glomerular basement membrane and infiltration of inflammatory cells in the model group. SHYS treatment alleviated the pathological changes in DKD model rats (Figure 1(d)). Likewise, the HE staining score for kidney injury was higher in the model group compared with the control group, the HE score was decreased in the SHYS low-dose and SHYS high-dose groups (*P* < 0.05, *P* < 0.01, respectively) compared with the control group (Figure 1(e)). The Masson and Sirius Red staining showed increased collagen content in the kidney in DKD model rats, and SHYS treatment reduced the accumulation collagen content in the kidney (Figures 1(f)–1(i)).

### 3.2. Anti-Inflammatory and Antioxidative Effects of SHYS on DKD Model Rats

**Figure 2:**
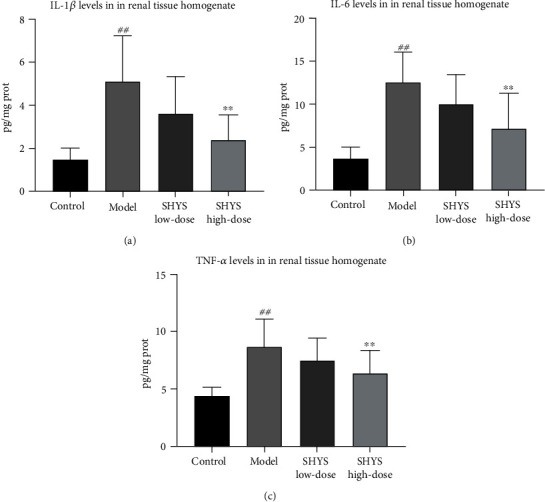
Anti-inflammatory effects of SHYS on DKD model rats. The anti-inflammatory effects of SHYS on DKD were evaluated by investigating the levels of proinflammatory cytokines (IL-1*β*, IL-6, and TNF-*α*) in renal tissue homogenates using ELISA. (a)–(c) SHYS treatment decreased the levels of IL-1*β* (a), IL-6 (b), and TNF-*α* (c) in renal tissue homogenates in DKD model rats.

The levels of proinflammatory cytokines (IL-1*β*, IL-6, and TNF-*α*) in renal tissue homogenate were detected using ELISA to evaluate the anti-inflammatory effects of SHYS on DKD model rats. Compared to the control group, the levels of IL-1*β*, IL-6, and TNF-*α* were significantly increased in the model group (*P* < 0.01, respectively). High-dose of SHYS treatment decreased the levels of IL-1*β*, IL-6, and TNF-*α* in the DKD model rats (*P* < 0.01, respectively, Figures 2(a)–2(c)).

The activities of SOD and GSH-Px and the level of MDA in renal tissue homogenate were also tested to study the effects of SHYS on oxidative stress in the DKD model rats. The results showed that the activities of SOD and GSH-Px were lower and the level of MDA was higher in the DKD model rats as compared with rats in control group (*P* < 0.01, respectively). The activity of SOD was increased in SHYS high-dose group compared to the model group (*P* < 0.05). Besides, low-dose and high dose of SHYS treatment decreased the MDA level (*P* < 0.05, *P* < 0.01, respectively) and increased the GSH-Px (*P* < 0.05, *P* < 0.01, respectively) activity in the DKD model rats (Table 3).

### 3.3. Effects of SHYS on PINK1/Parkin-Mediated Mitophagy in DKD Model Rats

**Figure 3:**
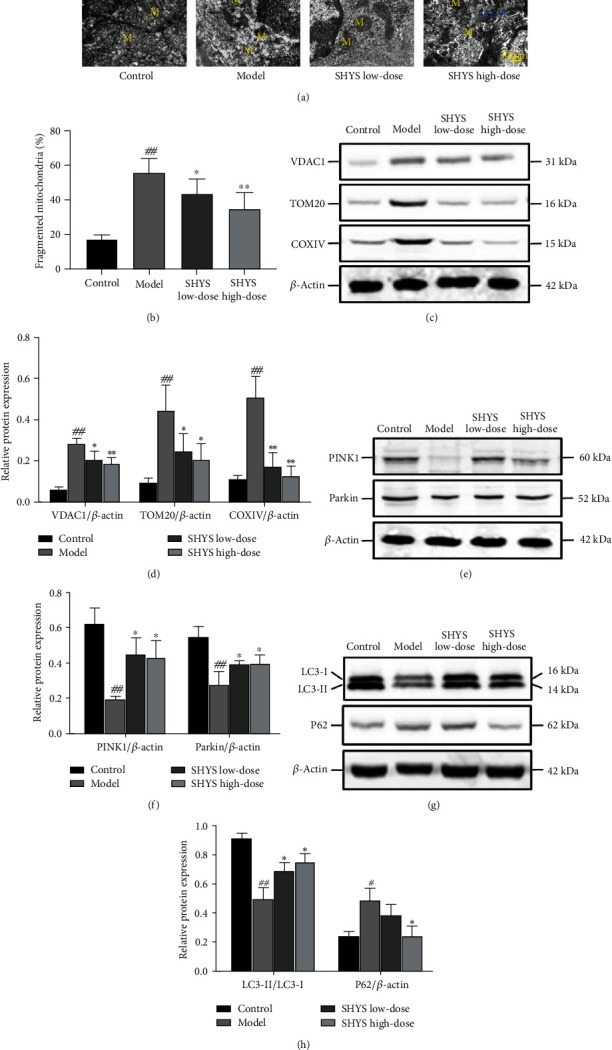
Effects of SHYS on PINK1/Parkin-mediated mitophagy in DKD model rats. The changes in morphology of the mitochondria in the kidney in each group were observed by transmission electron microscopy and the levels of proteins related to PINK1/Parkin-mediated mitophagy were detected by western blot. (a, b) Transmission electron microscopy showed that SHYS treatment improved the morphology of mitochondria in kidney (M indicated mitochondria and MS indicated mitophagosomes) (10,000×) (a). The percentage of fragmented mitochondria in transmission electron microscopy was calculated. SHYS treatment decreased the percentage of fragmented mitochondria in the kidney (b). (c, d) SHYS treatment decreased the levels of VDAC1, TOM20, and COXIV in kidney. (e, f) SHYS treatment increased the levels of PINK1 and Parkin in the kidney. (g, h) SHYS treatment increased the level of LC3-II and decreased the level of p62 in kidney. ^#^: *P* < 0.05 compared with the control group; ^##^: *P* < 0.01 compared with the control group; ^∗^: *P* < 0.05 compared with the model group; ^∗∗^: *P* < 0.01 compared with the model group.

Electron microscopy was used to observe the changes in morphology of mitochondria in the kidneys of each group. The membranes of mitochondria and the mitochondrial cristae in the control group were clear and distinct. The membranes of mitochondria in the model group were unclear and the mitochondrial cristae was lost. The structures of mitochondria exhibited much clarity in the SHYS low-dose and SHYS high-dose groups. In addition, isolation membranes and mitophagosomes could also be observed in the SHYS high-dose group (Figure 3(a)). The proportion of fragmented mitochondria was increased in the model group compared with the control (*P* < 0.01), whereas low-dose and high-dose of SHYS treatment decreased the proportion of fragmented mitochondria (*P* < 0.05, *P* < 0.01, respectively) in rats with DKD (Figure 3(b)).

Furthermore, the protein levels of mitochondrial membrane proteins (VDAC1, TOM20, and COXIV) in the kidney were increased in DKD model rats compared to the rats in the control group (*P* < 0.01, respectively). The protein levels of VDAC1 (*P* < 0.05, *P* < 0.01, respectively), TOM20 (*P* < 0.05, respectively), and COXIV (*P* < 0.01, respectively) were lower in the SHYS low-dose and SHYS high-dose groups as compared with those in the model group (Figures 3(c) and 3(d)). In addition, the protein levels of PINK1 and Parkin in the kidney were decreased in the model group compared to the control group (*P* < 0.01, respectively). Compared with the model group, low-dose and high-dose of SHYS treatment increased the protein levels of PINK1 (*P* < 0.05, respectively) and Parkin (*P* < 0.05, respectively) in the kidney (Figures 3(e) and 3(f)). The protein level of LC3-II (*P* < 0.01) was decreased and the protein level of p62 (*P* < 0.05) was increased in the model group compared with the control group. The protein level of LC3-II was higher in the SHYS low-dose and SHYS high-dose groups as compared to the model group (*P* < 0.05, respectively) and the protein level of p62 was lower in the SHYS high-dose group compared with the model group (*P* < 0.05, Figures 3(g) and 3(h)).

**Figure 4:**
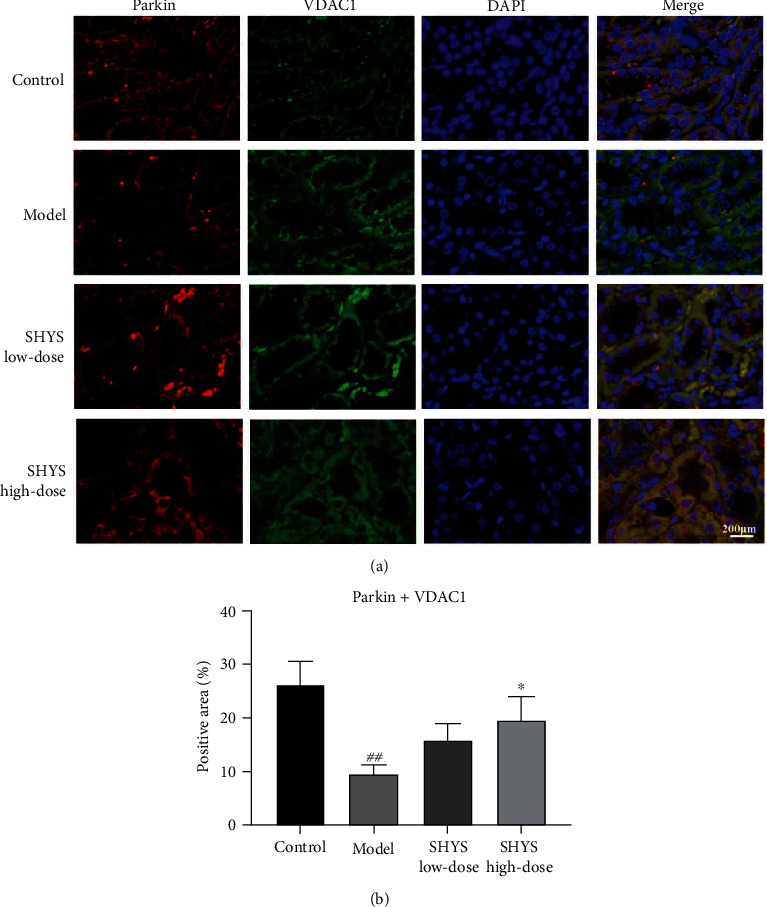
SHYS treatment increased the coexpression of Parkin and VDAC1 in DKD model rats. The coexpression of Parkin and VDAC1 in the kidney was investigated using immunoflourescence. (a) The mange of immunoflourescence staining in each group (400×). (b) Quantitative of immunoflourescence based on positive area.

**Figure 5:**
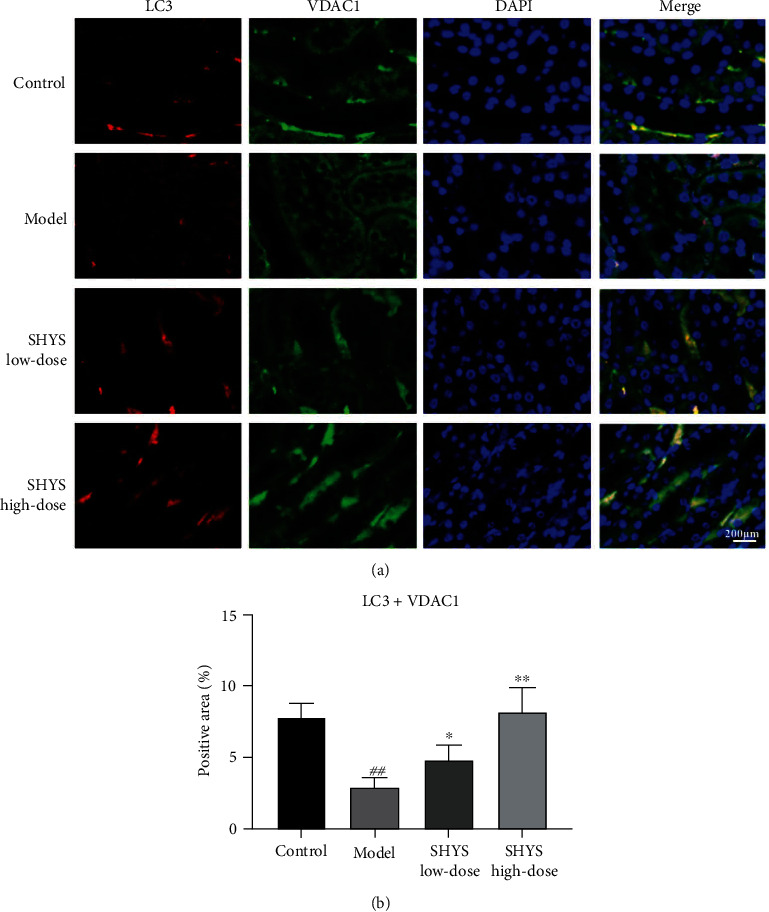
SHYS treatment increased the coexpression of LC3 and VDAC1 in DKD model rats. The coexpression of LC3 and VDAC1 in the kidney was investigated using immunoflourescence. (a) The mange of immunoflourescence staining in each group (400×). (b) Quantitative of immunoflourescence based on positive area.

**Figure 6:**
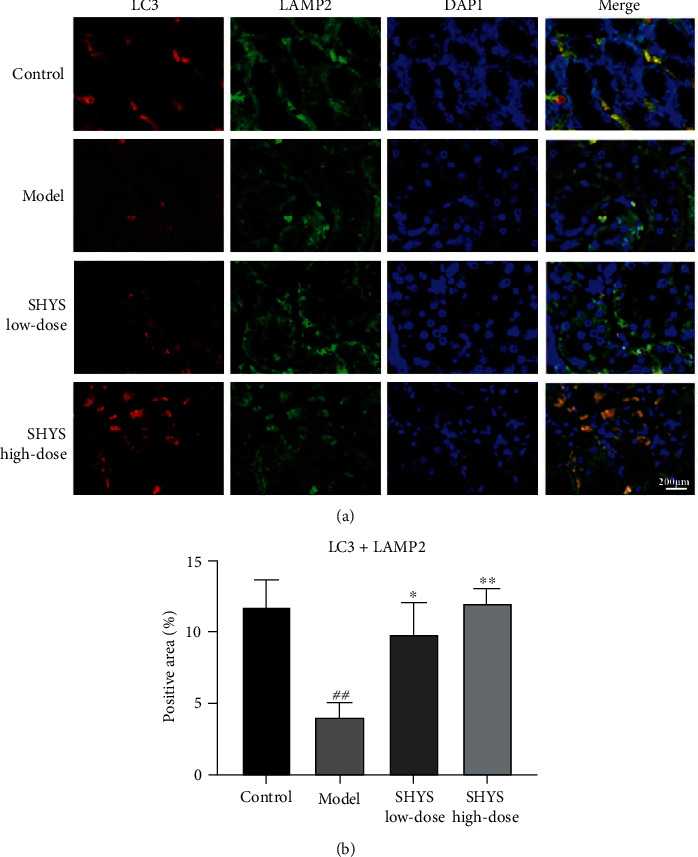
SHYS treatment increased the coexpression of LC3 and LAMP2 in DKD model rats. The coexpression of LC3 and LAMP2 in the kidney was investigated using immunoflourescence. (a) The mange of immunoflourescence staining in each group (400×). (b) Quantitative of immunoflourescence based on positive area.

Besides, immunoflourescence staining showed that the coexpressions of Parkin and VDAC1, LC3 and VDAC1, as well as LC3 and LAMP2, in the kidney were lower in the model group compared with the control group (*P* < 0.01, respectively). The coexpression of Parkin and VDAC1 (*P* < 0.05, Figures 4(a) and 4(b)) was increased in SHYS high-dose groups as compared to the model group. The coexpression of LC3 and VDAC1 (*P* < 0.05, *P* < 0.01, respectively, Figures 5(a) and 5(b)) as well as LC3 and LAMP2 (*P* < 0.05, *P* < 0.01, respectively, Figures 6(a) and 6(b)) in the kidney were increased in the SHYS low-dose and SHYS high-dose groups as compared to the model group.

### 3.4. Effects of SHYS on NLRP3 Signaling Pathway in DKD Model Rats

**Figure 7:**
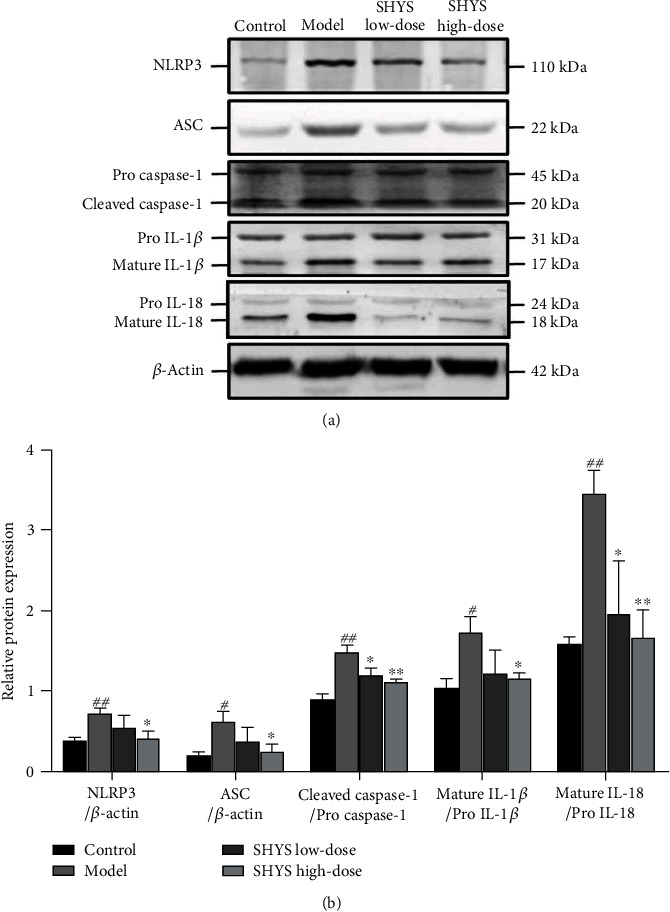
SHYS treatment inhibited the activation of NLRP3 signaling pathway in DKD model rats. The levels of proteins related to NLRP3 signaling pathway were detected by western blot. (a) Relative image of western blot (b) Analysis of relative protein expression based on gray values.

The activation of NLRP3 signaling pathway in the kidney in DKD model rats after SHYS treatment was evaluated using western blot. Compared with the control group, the protein levels of NLRP3 (*P* < 0.01), ASC (*P* < 0.05), cleaved caspase-1 (*P* < 0.01), mature IL-1*β* (*P* < 0.05), and mature IL-18 (*P* < 0.01) were increased in the model group. The protein levels of NLRP3, ASC, and mature IL-1*β* were lower in the SHYS high-dose group as compared to the model group (*P* < 0.05, respectively). The protein levels of cleaved caspase-1 (*P* < 0.05, *P* < 0.01, respectively) and mature IL-18 (*P* < 0.05, *P* < 0.01, respectively) were lower in the SHYS high-dose group as compared to the model group (Figures 7(a) and 7(b)).

## 4. Discussion

In this study, we first used a high-fat diet combined with STZ injection to construct a rat model of DKD. Compared to the control group, the FBG level in the model group significantly increased. The rats showed renal function-related biochemical marker abnormalities, presenting as elevated serum Cr and BUN and a significant increase in 24-hour urine protein level. In addition, pathological studies showed that renal tissues from the DKD model rats exhibited significant renal tubule atrophy, glomerular hyperplasia, and inflammatory cell infiltration. These pathological changes were consistent with DKD's pathological manifestation [12]. SHYS treatment decreased FBG and ameliorated the body weight loss in DKD rats, improved renal function-related biochemical markers, and reduced pathological changes in renal tissues to varying degrees, showing that SHYS has therapeutic effects in DKD, which was most significant in the high-dose SHYS group. IR is also an important pathological process during the progression of DKD [13]. We tested the HOMA-IR to study the effects of SHYS on IR. Results showed that SHYS-treated rats exhibited lower HOMA-IR compared with rats in the model group.

We further examined the effects of SHYS on inflammation and oxidative stress in DKD rats. Hyperglycemia can cause chronic inflammation, promote infiltration of inflammatory cells in renal tissues, and produce large amounts of proinflammatory factors, such as IL-1*β*, IL-6, and TNF-*α* [14]. The release of these inflammatory factors damages renal glomerular mesangial cells, epithelial cells, and renal interstitium, aggravating proteinuria [15]. Many studies have shown that the levels of inflammatory factors are positively correlated with the severity of DKD [16]. Our results showed that SHYS has anti-inflammatory effects based on the levels of proinflammatory factors in the renal tissues. Oxidative stress is an important pathological response during the progression of DKD. Hyperglycemia can directly induce the production of cause reactive oxygen species (ROS) in the kidney. High levels of ROS could cause the damage in renal cells and impair the renal function in DKD. In addition, high levels of inflammatory factors can also contribute to the accumulation of ROS [17]. Our results indicated that SHYS can upregulate sodium oxide dismutase (SOD) and GSH-Px activity and downregulate MDA levels in renal tissues. Malondialdehyde (MDA) is a cytotoxic lipid peroxidation product, and its level is positively correlated with oxidative stress [18]. SOD and glutathione peroxidase (GSH-Px) are both antioxidant enzymes that participate in ROS scavenging [19]. SOD can promote ROS conversion to H_2_O_2_, which then undergoes catalysis by GSH-Px to become water and oxygen. Increasing SOD and GSH-Px activity can protect renal tissues from oxidative stress-induced damage [20].

In addition, we studied the effects of SHYS on the morphology of mitochondria in renal tissues of DKD model rats. Electron microscope showed accumulated damage in the mitochondria in the kidney in DKD model rats, and SHYS treatment ameliorated the damage of mitochondrial crista and mitochondrial membrane in the kidney in DKD model rats. Dysregulated glucose metabolism is a pathophysiological change in DKD and is the primary factor inducing DKD occurrence and progression [21]. Persistent hyperglycemia-induced oxidative stress can cause damage to the mitochondrial outer membrane, resulting in depolarization changes in the membrane potential, causing oxidative damage to tissues and organs, particularly to the mitochondria. The accumulation of damage in the mitochondria further damages the innate cells of the kidneys, including podocytes, glomerular endothelial cells, mesangial cells, and renal tubule epithelial cells [22].

Therefore, we studied the effects of SHYS on mitophagy in DKD rats. Mitophagy is the cell's selective degradation and clearance of damaged or excess mitochondria through autophagy to maintain stable mitochondrial quantity and quality [23]. Our results demonstrated that SHYS can decrease VDAC1, Tom20, and COXIV expression in DKD rat renal tissues. VDAC1 and Tom20 are mitochondrial outer membrane proteins, and COXIV is an essential enzyme in mitochondrial oxidative phosphorylation [24]. Studies have shown that VDAC1, Tom20, and COXIV expression is upregulated when mitophagy is blocked, and VDAC1, Tom20, and COXIV expression gradually decreases when mitophagy increases.

Recent studies have shown that PINK1/Parkin-mediated mitophagy is inseparable from DKD. The in vivo and in vitro experiments proved that PINK1/Parkin-mediated mitophagy is inhibited in renal tubule epithelial cells in the DKD model, and activating PINK1/Parkin-mediated mitophagy can significantly improve mitochondrial function in renal tissues to alleviate kidney injury in DKD [24]. Our results indicated that SHYS can increase PINK1 and Parkin expression in DKD rat renal tissues. The serine/threonine kinase PINK1 is a molecular sensor of damaged mitochondria and is a starting signal for mitophagy [25]. When mitochondrial function is normal, PINK1 can enter the mitochondria through the mitochondrial membrane and undergo degradation, thereby maintaining a low level in the cytoplasm. PINK1 cannot be degraded when the mitochondria are damaged and stably accumulate in the outer mitochondrial membrane [26]. High levels of PINK1 can phosphorylate the E3 ubiquitin ligase Parkin to promote Parkin translocation into the mitochondria [27]. We used immunofluorescence staining to observe the colocalization levels of Parkin and the mitochondrial membrane protein VDAC1. Results showed that Parkin and VDAC1 colocalization levels significantly increased in rat renal tissues after SHYS intervention, suggesting that SHYS can promote Parkin accumulation in the mitochondria. These results indicate that SHYS can enable the initiation of PINK1/Parkin-mediated mitophagy.

We also measured the effects of SHYS on mitophagy flux, and the results showed that SHYS intervention could decrease p62 expression and increase LC3-II levels in renal tissues. Moreover, immunofluorescence staining indicated that SHYS treatment increased the colocalization of LC3 and VDAC1 in kidney. After Parkin has accumulated in the mitochondria, it can ligate ubiquitin (Ub) to Ub substrate or mitochondrial outer membrane proteins. The ubiquitin chains formed can be selectively recognized by the autophagy receptor p62 [28]. p62 is a vital autophagy regulatory protein and increased p62 levels suggest that autophagy is blocked. p62 can interact with LC3 to bind to it so that mitophagosomes and lysosomes are fused to form mature mitochondrial autophagolysosomes and activate mitochondrial degradation, thereby removing damaged mitochondria and restoring cell metabolic equilibrium [29]. LAMP2 is an important lysosome marker and detecting LC3 and LAMP2 colocalization levels can reflect autophagosome and lysosome fusion [30]. Our results similarly showed that SHYS could increase LC3 and LAMP2 colocalization levels.

A recent study found that blockade of PINK1/Parkin-mediated mitophagy can induce NLRP3 inflammasome activation, and the activation of PINK1/Parkin-mediated mitophagy can block NLRP3 inflammasome activation [31]. Our results showed that SHYS can decrease NLRP3, ASC, caspase-1, IL-1*β*, and IL-18 expression in DKD rat renal tissues. Under physiological conditions, NLRP3 within cells is in an autoinhibitory state. Hyperglycemia can cause NLRP3 to recruit ASC and bind to pro-caspase-1 to form an inflammasome and activate caspase-1. Activated caspase-1 can cleave pro-IL-1*β* and pro-IL-18 to produce active IL-1*β* and IL-18, respectively, releasing from cells and worsening renal tissue inflammation.

In summary, our study demonstrated the therapeutic effects of SHYS on DKD. Additionally, our results indicated that SHYS promotes PINK1/Parkin-mediated mitophagy in renal tissues and inhibits NLRP3 inflammasome activation to improve mitochondrial injury and inflammatory responses.

## Figures and Tables

**Table 1:** Levels of Cr and BUN in serum after SHYS treatment.

Group	Cr (*μ* mol/L)	BUN (mmol/L)
Control	26.2 ± 16.43	3.64 ± 0.85
Model	88.08 ± 20.56^##^	7.27 ± 2.2^##^
SHYS low-dose	63.57 ± 15.89^∗^	5.94 ± 2.19
SHYS high-dose	49.88 ± 24.39^∗∗^	4.85 ± 2.1^∗^

Control, model, SHYS low-dose, SHYS high-dose groups (*n* = 10 per group) ^##^: *P* < 0.01 compared with the control group; ^∗^: *P* < 0.05 compared with the model group; ^∗∗^: *P* < 0.01 compared with the model group.

**Table 2:** Effects of SHYS on FINS and HOMA-IR.

Group	FINS (*μ* IU/ml)	HOMA-IR
Control	6.17 ± 1.22	1.66 ± 0.36
Model	13.21 ± 3.01^##^	17.40 ± 5.09^##^
SHYS low-dose	9.41 ± 2.69^∗^	9.22 ± 3.39^∗∗^
SHYS high-dose	8.24 ± 2.83^∗∗^	6.58 ± 2.88^∗∗^

**Table 3:** Activities of SOD and GSH-Px and level of MDA in renal tissue homogenates after SHYS treatment.

Group	SOD (U/mgprot)	MDA (nmol/mgprot)	GSH-Px (U/mgprot)
Control	177.05 ± 26.88	3.73 ± 0.70	84.11 ± 10.43
Model	121.56 ± 27.62^##^	14.96 ± 3.57^##^	40.79 ± 12.85^##^
SHYS low-dose	136.66 ± 35.73	11.48 ± 3.00^∗^	56.08 ± 15.54^∗^
SHYS high-dose	154.02 ± 31.93^∗^	10.26 ± 3.11^∗∗^	64.15 ± 19.39^∗∗^

## Data Availability

The datasets used and/or analysed during the current study are available from the corresponding author on reasonable request.

## Supplementary Materials

Supplementary Materials The HE staining score for kidney injury was shown in Table S1. Besides, The DKD rat model was verified based on the levels of blood glucose (Figure S1a) and 24 h of urine protein (Figure S1b). One week after the STZ injection, the BG in model rats was higher than 16.7 mmol/L and the urine protein level was above 20 mg/24 h.
