# Supplementary material for: San-Huang-Yi-Shen Capsule Ameliorates Diabetic Kidney Disease through Inducing PINK1/Parkin-Mediated Mitophagy and Inhibiting the Activation of NLRP3 Signaling Pathway
Source: J Diabetes Res. 2022 Nov 15;2022:2640209. doi: 10.1155/2022/2640209 (PMC9681560; doi:10.1155/2022/2640209)
Supplement: Supplementary Materials — The HE staining score for kidney injury was shown in Table S1. Besides, The DKD rat model was verified based on the levels of blood glucose (Figure S1a) and 24 h of urine protein (Figure S1b). One week after the STZ injection, the BG in model rats was higher than 16.7 mmol/L and the urine protein level was above 20 mg/24 h. [file 2640209.f1.docx]

**Table S1 HE staining score for kidney injury**

| **Score** | **Description** |
| --- | --- |
| 0 | No pathological changes can be observed in glomeruli and renal tubules. |
| 1 | Most of the glomeruli are normal, a few of them have mesangial hyperplasia, and there is no lesion in the renal tubules and renal interstitium. |
| 2 | <50% of glomeruli have mesangial hyperplasia and there is no lesion in the renal tubules and renal interstitium. |
| 3 | Grademost of the glomeruli have mesangial hyperplasia, accompanied by renal inter stitial damage. |
| 4 | Gradeall glomeruli have mesangial hyperplasia and sclerosis, accompanied byrenal interstitial damage. |
| 5 | Similar to the grade 4, but the damage of the renal tubules and renal interstitium is more severe than the grade 4. |

**a**


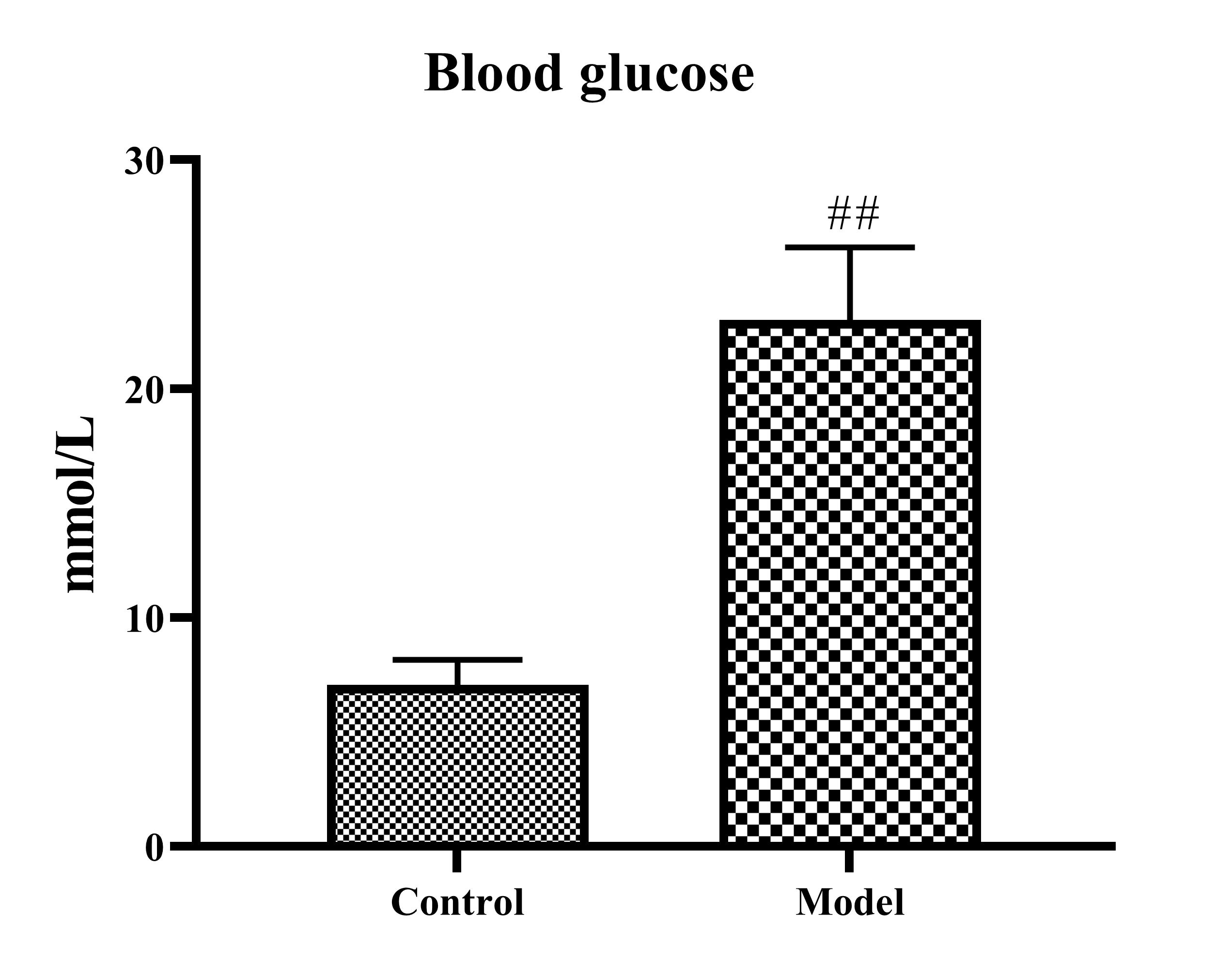


**b**


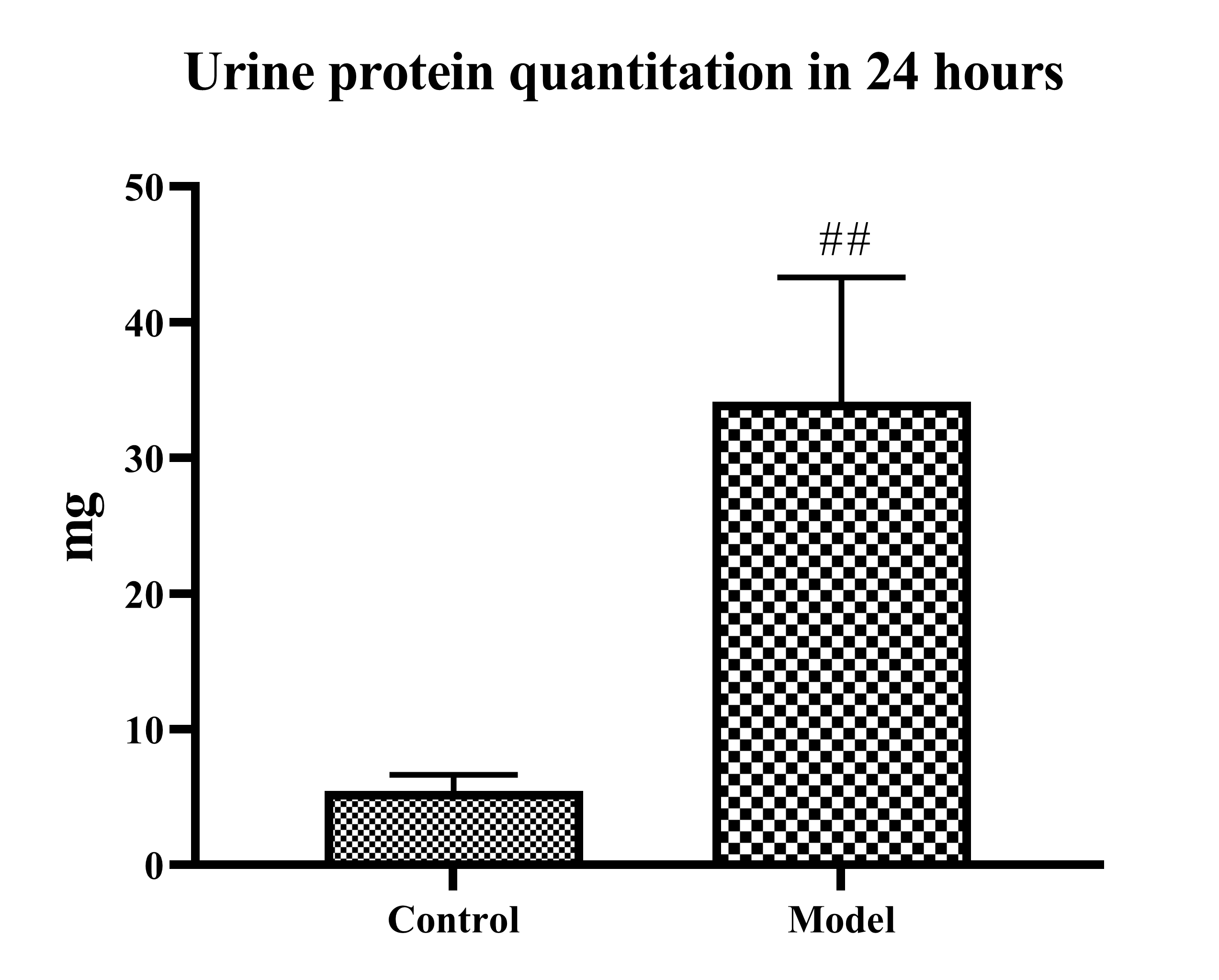


**Figure S1:** The DKD rat model was verified based on the levels of blood glucose (**a**) and 24 h of urine protein (**b**). One week after the STZ injection, the BG in model rats was higher than 16.7mmol/L and the urine protein level was above 20mg/24h. Control group (n=10), Model group (n=30) ^##^: *P* < 0.01 compared with the control group
